# Galanin-Mediated Behavioural Hyperalgesia from the Dorsomedial Nucleus of the Hypothalamus Involves Two Independent Descending Pronociceptive Pathways

**DOI:** 10.1371/journal.pone.0142919

**Published:** 2015-11-13

**Authors:** Diana Amorim, Hanna Viisanen, Hong Wei, Armando Almeida, Antti Pertovaara, Filipa Pinto-Ribeiro

**Affiliations:** 1 Life and Health Sciences Research Institute (ICVS), University of Minho, Braga, Portugal; 2 ICVS/3B’s—PT Government Associate Laboratory, Braga/Guimarães, Portugal; 3 Institute of Biomedicine/Physiology, University of Helsinki, Helsinki, Finland; Boston Children’s Hospital and Harvard Medical School, UNITED STATES

## Abstract

Activation of the dorsomedial nucleus of the hypothalamus (DMH) by galanin (GAL) induces behavioural hyperalgesia. Since DMH neurones do not project directly to the spinal cord, we hypothesized that the medullary dorsal reticular nucleus (DRt), a pronociceptive region projecting to the spinal dorsal horn (SDH) and/or the serotoninergic raphe-spinal pathway acting on the spinal 5-HT_3_ receptor (5HT_3_R) could relay descending nociceptive facilitation induced by GAL in the DMH. Heat-evoked paw-withdrawal latency (PWL) and activity of SDH neurones were assessed in monoarthritic (ARTH) and control (SHAM) animals after pharmacological manipulations of the DMH, DRt and spinal cord. The results showed that GAL in the DMH and glutamate in the DRt lead to behavioural hyperalgesia in both SHAM and ARTH animals, which is accompanied particularly by an increase in heat-evoked responses of wide-dynamic range neurons, a group of nociceptive SDH neurones. Facilitation of pain behaviour induced by GAL in the DMH was reversed by lidocaine in the DRt and by ondansetron, a 5HT_3_R antagonist, in the spinal cord. However, the hyperalgesia induced by glutamate in the DRt was not blocked by spinal ondansetron. In addition, in ARTH but not SHAM animals PWL was increased after lidocaine in the DRt and ondansetron in the spinal cord. Our data demonstrate that GAL in the DMH activates two independent descending facilitatory pathways: (i) one relays in the DRt and (ii) the other one involves 5-HT neurones acting on spinal 5HT_3_Rs. In experimental ARTH, the tonic pain-facilitatory action is increased in both of these descending pathways.

## Introduction

Galanin (GAL) inhibits or enhances nociceptive transmission in the spinal cord depending on (i) its concentration, with high concentrations being antinociceptive and low concentration pronociceptive [1] and (ii) the availability of its receptors, with GALR1/3 being inhibitory and GALR2 facilitatory [1]. Although supraspinal GAL was considered antinociceptive [2–5], it was recently shown that its administration to the dorsomedial nucleus of the hypothalamus (DMH) induces heat hyperalgesia [6]. Moreover, GAL/DMH-mediated pronociception was shown to be dependent on the activation of GALR1 [6]. Interestingly, DMH activation by glutamate or disinhibition by bicuculline induced behavioural hyperalgesia mediated by the rostral ventromedial medulla (RVM) in controls [7,8] but not in monoarthritic animals [8]. However, when GAL was microinjected in the DMH, both control and monoarthritic animals displayed behavioural hyperalgesia, although this effect could not be correlated to the activity of RVM On- and Off-like cells involved in descending modulation of nociception [6]. These findings leave open which relay nuclei and descending pathways mediate the pronociception induced by GAL in the DMH.

Since DMH neurones do not project directly to the spinal cord [9,10], a potential pronociceptive pathway mediating the descending action of GAL in the DMH is the dorsal reticular nucleus (DRt) [11], a structure receiving strong afferent projections from the DMH [9,10]. Neurones in the DRt respond exclusively or mainly to noxious stimulation [12,13]. DRt lesion decreased hyperalgesia in the acute and late phase of the formalin test [14] and reduced the noxious heat-evoked behavioural responses [15]. In line with this, Dugast and colleagues [16] demonstrated that activation of the DRt enhanced the noxious stimulation-evoked activity of spinal nociceptive neurones. Although the role of the DRt in chronic pain is poorly understood, there is evidence suggesting that the DRt contributes to the maintenance of central sensitization in experimental neuropathy [17], and this DRt-induced facilitatory effect is modulated by opioids [18] and noradrenaline [19].

In our recent study [6], GAL in the DMH induced behavioural hyperalgesia without an accompanying increase in the activity of presumably pronociceptive RVM On-cells or a decrease in the activity of antinociceptive RVM Off-cells, which are changes associated with pronociception [8,20–23]. However, the RVM has a subpopulation of serotoninergic neurones that are not On- or Off-cells and which potentially contribute to descending regulation of nociception through action on spinal 5-HT receptors [24]. Spinally projecting serotonergic pathways are reported to enhance or inhibit nociception [25,26] and the direction of effect has varied from antinociception to pronociception with the subtype of 5-HT receptor. Suzuki and colleagues [27] showed that the activation of spinal serotonin type-3 receptors (5HT_3_R) enhanced nociception in chronic pain states. Earlier results [6] leave open the possibility that serotoninergic pathways originating in the RVM and acting on the spinal 5HT_3_R contribute to the hyperalgesia induced by GAL in the DMH.

Here we tested the hypotheses that the hyperalgesic effect of GAL in the DMH is mediated by the DRt and/or descending serotoninergic pathways acting on spinal 5-HT_3_Rs. Experiments were performed in healthy controls and animals with experimental monoarthritis.

## Methods

### Animals, ethical issues and anaesthesia

The experiments were performed using adult male Hannover–Wistar rats (220–260 g; n = 12, Harlan, Horst, The Netherlands) in the Biomedicum Helsinki, Finland. All experiments were approved by the ethical committee for experimental animals studies of the State Provincial Office of Southern Finland (Hämeenlinna, Finland) and the experiments were performed according to the guidelines of European Communities Council Directive of 22^nd^ September 2010 (2010/63/EU). All efforts were done to minimize animal suffering and to reduce the number of animals used.

The animals were housed in polycarbonate cages with a deep layer of saw dust, one animal in each cage, in a thermo-statically controlled room at 24.0 ± 0.5°C. The room was artificially illuminated from 8.30 a.m. to 8.30 p.m. The animals received commercial pelleted rat feed (CRM-P pellets, Special Diets Services, Witham, Essex, England) and water *ad libitum*.

The anaesthesia, for surgeries and electrophysiological sessions, was induced by administering pentobarbitone (60 mg/kg, intraperitoneal; OrionPharma, Espoo, Finland). The level of anaesthesia was monitored by verifying behavioural responses to noxious pinching. If the pinch-evoked response was stronger than a brief muscle twitch restricted to the stimulated limb, the animal was given an additional dose of pentobarbitone (15–20 mg/kg, intraperitoneal).

### Induction of monoarthritis

The induction of monoarthritis (ARTH) was performed, under anaesthesia, four weeks before the behavioural experiments, as described elsewhere [8]. Briefly, 3% kaolin and 3% carrageenan (Sigma-Aldrich, St. Louis, MO, USA) were dissolved in sterile saline solution (0.9% NaCl) and injected into the synovial cavity of the right knee joint at a volume of 0.1 mL. This model produces mechanical hyperalgesia, which begins just a few hours after kaolin/carrageenan (K/C) injection and lasts for several weeks [28]. Control animals (SHAM) were injected with 0.1 mL saline in the synovial cavity of the right knee joint.

In each animal, the development of monoarthritis was verified 1h prior to each experiment. Only those rats that vocalized every time during five flexion–extension movements of the knee joint were considered to have developed monoarthritis and were included in the ARTH group. SHAM animals did not vocalize to any of the five consecutive flexion–extension movements of the knee joint.

In order for the experimenter to remain blinded in relation to which animals were SHAMs or ARTHs the identification of the animals cages gave no clue as to whether the animals belonged to the SHAM or ARTH group. The identification codes revealing the experimental condition what groups the animals belonged to as was only attributed a number and the correspondence codes were held by a third party. After four weeks of arthritis ARTH animals do not display any evident alterations in spontaneous behaviour that would allow the experimenter to distinguish between ARTH and SHAM animals.

### Surgical procedures for the installation of the intrathecal catheter

For intrathecal (i.t.) drug administration, a catheter (PE-10, Becton Dickinson and Company, Sparks, MD, USA) was implanted at the lumbar level of the spinal cord as described in detail elsewhere [29]. In the following day, the correct placement of the catheter was verified by administering lidocaine (7 μL, 4%; OrionPharma, Espoo, Finland) with a 50 μl Hamilton syringe (Hamilton Company, Bonaduz, Switzerland). Only those rats that had no motor impairment before lidocaine injection but had a bilateral paralysis of hind limbs following i.t. administration of lidocaine (LIDO) were further studied. During the pharmacological sessions, the drugs were microinjected i.t. with a 50 μL Hamilton syringe in a volume of 5 μL. To minimize the number of times the animal was anesthetized, the installation of the i.t. catheter and the induction of monoarthritis were performed at the same time.

### Surgical procedures for intracerebral cannula implantation

For intracerebral drug administration, two stainless steel guide cannulae (26 gauge; PlasticsOne, Roanoke, USA) were implanted in the DMH and DRt of each animal, according to the coordinates of the rat brain atlas [30]. The tips of the guide cannulae were positioned 1 mm above the desired injection site in the DMH [anteroposterior (AP), -3.24 mm from bregma; lateromedial (LM), 0.4 mm lateral from the midline (on the right side); dorsoventral (DV), 7.5 mm below the surface of the skull] and the DRt [AP, -14.04 mm from bregma; LM, 1.8 mm lateral from the midline (on the right side); DV, 7.6 mm below the surface of the skull]. The guide cannulae were kept in place with dental screws and dental cement. A dummy cannula was inserted into each guide cannula to prevent their blockade. The animals were allowed to recover from the surgery for at least one week after the behavioural tests were performed.

Test drugs were administered in the DMH and in the DRt through a 33-gauge injection cannula (PlasticsOne) with an injection volume of 0.5 μL. The spread of the injected drugs within the brain was expected to have a diameter of at least 1 mm [31]. The efficacy of injections was monitored by watching the movement of a small air bubble through the tubing. The injection lasted 20 s and the injection cannula was left in place for additional 30 s to minimize the return of drug solution back to the injection cannula.

### Pressure application measurement

The application of noxious pressure to the primary site of injury is a classical approach to measure mechanical hyperalgesia [32], both in humans and animals [33]. Here, a new pressure application measurement (PAM) method was used to evaluate mechanical hyperalgesia after four weeks of ARTH induction. It allows an accurate behavioural measurement of primary mechanical hypersensitivity in rodents with chronic inflammatory joint pain [34] by the application of a force range of 0–1500 g. Before the beginning of the test the experimenter practiced the correct application of the desired force by following a linear graph provided by the PAM software. To perform the test and with the animal securely held, the force transducer unit (fitted to the experimenter’s thumb) is placed on one side of the animal's knee joint and the forefinger on the other. Then, an increasing force is applied across the joint at a rate of approximately 300 g/s, as defined in the software, until a behavioural response is observed (limb-withdrawal, freezing of whisker movement, wriggling or vocalization) with a cut-off of 5 s. The peak force applied immediately prior to the behavioural response is recorded, by the real-time measurements system of the PAM software, as the limb withdrawal threshold (LWT). Three measurements of the ipsilateral and contralateral limbs were made at 1 min intervals. The mean LWTs were calculated per animal.

### Drugs

Solutions for intracerebral drug injections in the DMH and DRt were prepared with sterilized saline 0.9% (Unither, Amiens, France; pH 7.2) except for LIDO, which was acquired as a solution. Each injection had a volume of 0.5 μL and contained either 50 nmol of GLU (Merck, Darmstadt, Germany), LIDO 4% (OrionPharma, Espoo, Finland), 1 nmol of GAL (Tocris, Bristol, UK) or 0.3 nmol ondansetron (OND) (Tocris). In the results section, the behavioural and electrophysiological changes induced by a drug were considered at the time point of maximum effect of each drug: 1 min for GLU, 20 min for GAL and LIDO and 30 min for OND.

### Course of the behavioural and pharmacological study

Rats were habituated to the experimental conditions by allowing them to spend 1–2 h daily in the experimental room during the three days preceding any testing. For assessing nociception in awake animals, radiant heat-induced latencies for withdrawing the hind paw (The plantar test instrument model 37370, Ugo Basile, Varese, Italy) were determined. In each pharmacological session paw-withdrawal latency (PWL) was assessed prior to drug administration and at the time of maximum effect of each drug according to previous studies [4,5,8,35]. The behavioural study started four weeks after the induction of ARTH and at least one week following the insertion of the guide cannulae in the DMH and DRt. Hind paw skin temperature was assessed with an electronic thermometer (BAT-12, Physitemp Instruments Inc., Clifton, NJ, USA) before and after drug administration in the DMH in order to exclude the confounding influence of changes in skin temperature upon PWL.

To study the effect of galanin (GAL) in the DMH upon the DMH-DRt-spinal cord pathway several pharmacological approaches were used: i) GAL administration in the DMH; ii) glutamate (GLU) administration in the DRt; iii) lidocaine (LIDO) administration in the DRt; iv) ondansetron (OND) administration in the spinal cord; v) LIDO in the DRt followed immediately by GAL in the DMH; vi) OND in the spinal cord followed 10 min later by GAL in the DMH and; vii) OND in the spinal cord followed 30 min later by GLU in the DRt. Saline solution (SAL) was used in control injections. All animals underwent all the treatment combinations. Different treatment combinations were studied in a random order in each animal. The minimum interval in testing different drug treatments in the same animal was 72 h.

The effect of a drug upon PWL was calculated as the difference between the value of PWL at the time of maximum effect of a drug and the value of PWL before drug administration; i.e., positive values represent an increase and negative ones a decrease in the PWL induced by the drug. ΔPWL = PWL (max effect)–PWL (baseline).

### Course of the electrophysiological recordings in the spinal dorsal horn

Electrophysiological recordings of spinal dorsal horn (SDH) neurones were performed in anaesthetized rats. Lumbar vertebrae T12-L2 were located, a laminectomy was performed, and segments L4–L6 of the spinal cord were exposed. The dura was removed and dehydration of the spinal cord was prevented with mineral oil. The animal was placed in a standard stereotaxic frame and two guide cannulae were implanted in the DMH and DRt as described above (section 2.4). To stabilize the spinal cord two spinal clamps, one rostral and one caudal to the laminectomy, were used. Extracellular recordings of dorsal horn neurones, ipsilateral to the arthritic knee or SAL-injected knee, were performed using lacquer-coated tungsten electrodes (tip impedance 3–10 MΩ at 1 kHz). The signal was amplified and filtered using standard techniques. Data sampling was performed with a computer connected to a CED Micro 1401 interface and using the Spike 2 software (Cambridge Electronic Design, Cambridge, UK). In the spinal dorsal horn, search and classification of spinal units was performed as described elsewhere [36]. The recording depth from the spinal cord surface was 0.4–1.0 mm [37] and all the spinal neurones included in the study had their receptive field in the plantar skin of the right hind paw. The evaluation of the electrophysiological properties of a SDH neurone consisted of the following assessments performed sequentially: i) spontaneous activity; ii) response to brushing 5 times (1 Hz); and iii) response to heating with a feedback-controlled contact thermostimulator, a heat ramp rising at the rate of 10°C/s from the baseline temperature of 35°C to the peak temperature of 54°C and peak duration of 10s after which the temperature was decreased to the baseline temperature of 35°C at a rate of 4°C/s (82.8 mm^2^, LTS-3 Stimulator, Thermal Devices Inc., Golden Valley, MN, USA) of the ipsilateral hind paw. Body temperature was maintained within a physiological range with a Homeothermic Blanket System (Harvard Apparatus Ltd, Edenbridge, U.K.).

The neurones activated by innocuous brush were classified as wide-dynamic range (WDR) neurones, while neurones responsive only to noxious heat stimulation were classified as nociceptive specific (NS) according to the criteria described earlier [37]. Spontaneous activity of neurones was recorded over a period of at least 30 min or until response stabilization. When analysing (before drug administration) the baseline properties of neurones, the response of a neurone was defined as the difference between neuronal activity during peripheral stimulation and the spontaneous activity of the neurone, i.e., positive values represent excitatory responses evoked by peripheral stimulation and negative ones inhibitory responses. The effect of a drug upon the spontaneous activity of a neurone was calculated as the difference between spontaneous activity at the time of maximum effect of a drug and the spontaneous activity before drug administration (baseline spontaneous activity); i.e., positive values represent an increase and negative ones a decrease of the spontaneous activity induced by the drug. The effect of a drug upon the peripheral stimulation-evoked response is the difference between the neuronal response to a stimulus at the time of maximum effect of a drug and the response to a peripheral stimulation before drug administration (baseline response); i.e., positive values represent a drug-induced increase and negative ones a decrease of the stimulus-evoked response. In the electrophysiological sessions, 3 intracerebral drug injections were performed in a random order in each animal. The interval between the end of the effect of a drug and the injection of the following drug was at least 1h. Spontaneous discharge rate before injection of each drug treatment was used as an intrinsic control for stability of neuronal activity between injections.

### Histology

**Fig 1:**
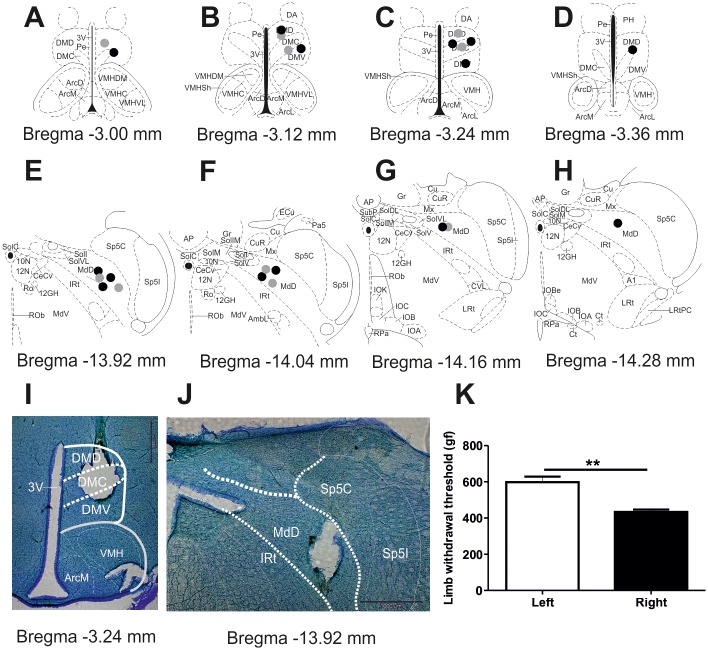
Anatomical confirmation of drug injection sites. Schematic representation of the injection sites in the dorsomedial nucleus of the hypothalamus (DMH) (**A-D**: -3.00 mm, -3.12 mm, -3.24 mm and -3.36 mm from bregma) and medullary dorsal reticular nucleus (DRt) (**E-H**: -13.92 mm, -14.04 mm, -14.16 and -14.28 mm from bregma) of SHAM (light dots; n = 7) and ARTH (dark dots; n = 5). Photomicrograph of an example of the drug injection site in the DMH (**I**: -3.24 mm from bregma) and the DRt (**J**: -14.16 mm from bregma). Limb withdrawal threshold four weeks after the induction of monoarthritis in the right knee, arthritic animals display a decreased limb withdrawal threshold in the right hind limb in the pressure application measurement test when compared to the left hind limb (**K**). Limb withdrawal threshold is presented as mean + SEM. (**P<0.01, t-test for paired data). gf—gram force.

At the end of the experimental period, animals received a lethal dose of pentobarbitone and the brains were removed and preserved in 10% formalin. Coronal sections were cut in a vibratome and stained with cresyl violet. Injection sites were histologically verified and plotted on standardized sections adapted from Paxinos and Watson [30]. Injection sites in the DMH and DRt are shown in Fig 1A–1C.

### Statistical analysis

The GraphPad Prism^®^ 6 (GraphPad Software Inc, La Jolla, CA, USA) and IBM^®^ SPSS^®^ Statistics 20.0 (IBM Corp, Armonk, NY, USA) software were used to perform the statistical analyses. The comparison of differences between sides in the PAM and heat-evoked paw withdrawal test and between the effects of SAL or GAL administration in the DMH on the hind paw skin temperature were performed using a Student’s t-test for paired data. Student’s t-test for unpaired data was used to compare the baseline PWL of the right (ipsilateral) hind paws, the spontaneous activity and heat-evoked activity of spinal neurones between SHAM and ARTH groups. One sample t-test was used to compare the mean variation of spontaneous and heat-evoked activities of WDR and NS neurones before and after GAL in the DMH or GLU in the DRt. The drug-induced differences in PWL between SHAM and ARTH groups were analysed using a two-way mixed ANOVA followed by a t-test with a Bonferroni correction for multiple comparisons. Statistical significance was accepted for *P* < 0.05. Data in the results section are expressed as mean + standard deviation of the mean (SD).

## Results

### Behavioural evaluation of nociception

#### Kaolin/carrageenan treatment induced monoarthritis

Three days after the intrasynovial injection of K/C, all animals in the ARTH group developed a clear swelling of the treated right knee joint and all vocalized during a minor extension and flexion of the affected limb by the experimenter. SHAM animals displayed no obvious swelling of the knee joint and did not vocalize when the limb was extended.

#### Arthritic animals display ipsilateral mechanical hyperalgesia

Mechanical hyperalgesia in the knee joint was assessed by determining LWT during the application of pressure to the knee joint. LWT of ARTH animals was significantly different between the left (598.3 ± 69.0) and the right (433.7 ± 29.3) knee joints (*t*_4_ = 5.845, *P* = 0.004), with the K/C injected right knee showing a significant decrease in the LWT, i.e., mechanical hyperalgesia.

#### The pronociceptive effect of GAL in the DMH is at least partly mediated by the DRt

Baseline PWL evoked by noxious heating of the ipsilateral hind paw (i.e., distal to the inflamed knee joint in the ARTH group) was not significantly different between SHAM and ARTH animals (*t*_10_ = 1.543, *P* = 0.154; SHAM = 7.0 ± 0.7 s, ARTH = 6.3 ± 0.8 s). Baseline PWL remained stable throughout the experimental period in both SHAM and ARTH animals (SHAM: F_GG3.057,18.34_ = 1.923, P = 0.161 and ARTH: SHAM: F_GG2.248,8.991_ = 1.928, P = 0.199).

**Fig 2:**
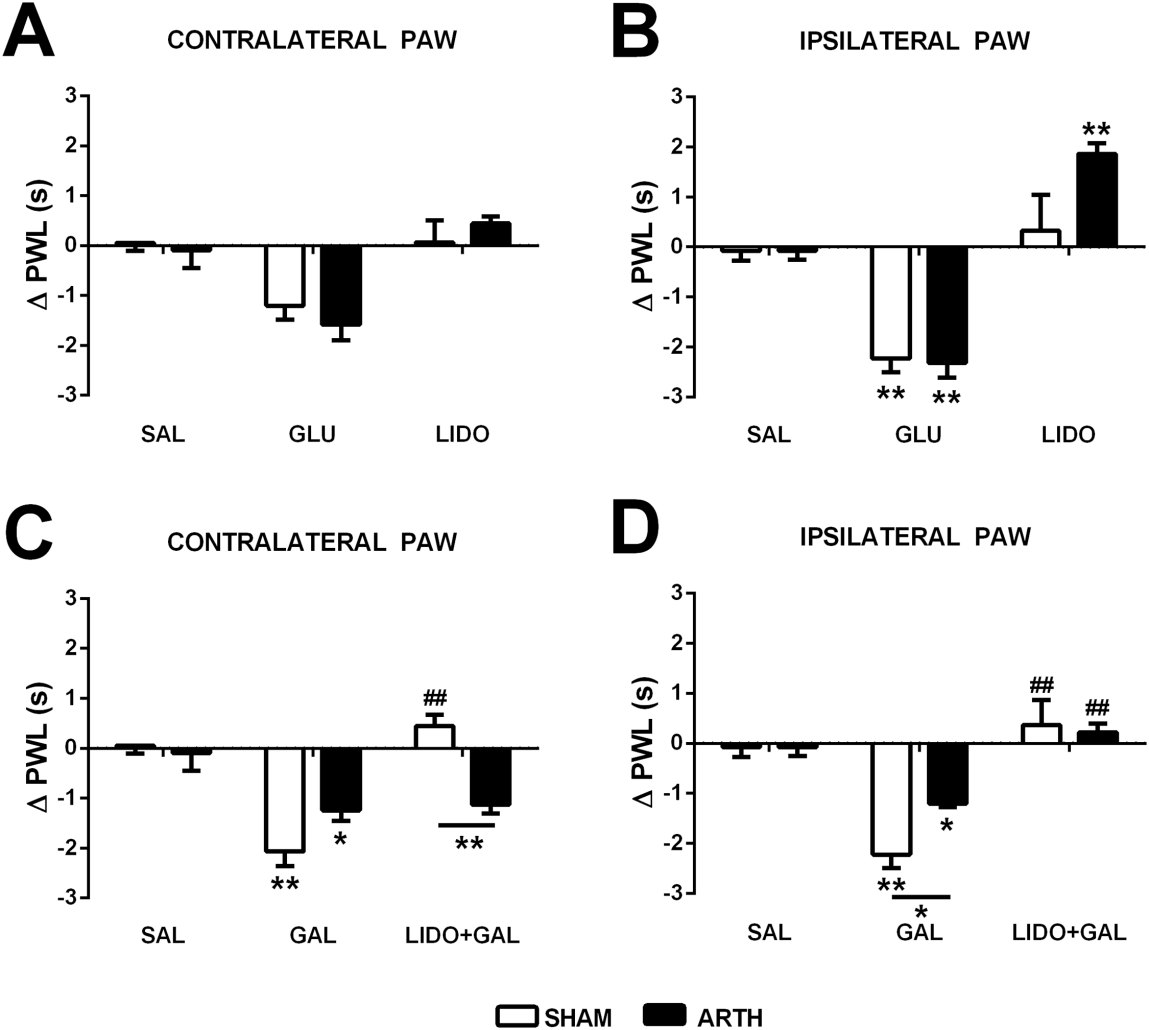
Behavioural evaluation of paw-withdrawal latencies (PWL) after drug microinjections in the dorsomedial nucleus of the hypothalamus (DMH) and the medullary dorsal reticular nucleus (DRt) of control (SHAM) and arthritic (ARTH) animals. PWL in the contralateral **(A)** and ipsilateral **(B)** hind limbs after glutamate (GLU) or lidocaine (LIDO) in the DRt. PWL in the contralateral **(C)** and ipsilateral **(D)** hind limbs after galanin (GAL) in the DMH or the combination of GAL in the DMH and LIDO in the DRt. Saline (SAL) injections were used as controls. Graphs show mean PWL + SEM (n_SHAM_ = 7; n_ARTH_ = 5). (*P<0.05, **P<0.01: Comparison of the drug effect with the effect of SAL injection in the same experimental group;^**##**^ *P*<0.01: Comparison of the effect of drug combination (GAL+LIDO) with the corresponding GAL alone group (t-test with a Bonferroni correction for multiple comparisons).

To study the phasic and tonic action of the DRt upon nociceptive modulation, PWLs on both contralateral and ipsilateral hind paws were evaluated after injecting GLU, LIDO or SAL in the right (ipsilateral to the inflamed knee joint in the ARTH group) DRt. In the contralateral (left) paw, drug injection in the DRt significantly altered PWL (main effect of drug: *F*_2,20_ = 7.388, *P* = 0.004), although post hoc tests did not indicate any difference between treatment groups (Fig 2A). In the ipsilateral (right) paw, drug injection in the DRt also significantly altered PWL (main effect of drug: *F*_2,20_ = 33.001, *P*<0.001) with *post hoc* tests indicating that PWL was significantly decreased after DRt activation by GLU in both SHAM and ARTH animals, and increased after DRt inhibition by LIDO in ARTH animals alone (Fig 2B).

To investigate a possible role of the (right) DRt in the mediation of the pronociceptive effect of GAL in the (right) DMH, PWL was assessed after inhibition of the DRt by LIDO followed by GAL microinjection in the DMH. In the contralateral (left) paw, drug injections significantly altered PWL (main effect of drug: *F*_2,20_ = 29.082, *P*<0.001) and this effect varied with the drug treatment (interaction between experimental group and drug treatment: *F*_2,20_ = 13.993, *P*<0.001). *Post hoc* tests indicated that PWL was decreased after GAL microinjection in the DMH in both SHAM and ARTH animals and that LIDO in the DRt reversed the GAL/DMH-induced effect in SHAM animals only (Fig 2C). In the ipsilateral (right) paw, drug injections significantly altered PWL (main effect of drug: *F*_2,20_ = 27.467, *P*<0.001). *Post hoc* testing indicated that PWL was decreased after GAL in the DMH in both SHAM and ARTH animals and that LIDO microinjection in the DRt was able to reverse this effect in both experimental groups (Fig 2D). Importantly, skin temperature of the hind paws did not vary with drug (SAL or GAL) administration in the DMH of SHAM (*t*_6_ = 0.819, *P* = 0.444) or ARTH animals (*t*_4_ = 0.270, *P* = 0.800).

#### The pronociceptive effect of GAL in the DMH is at least partly mediated by 5HT_3_R at the spinal cord level

**Fig 3:**
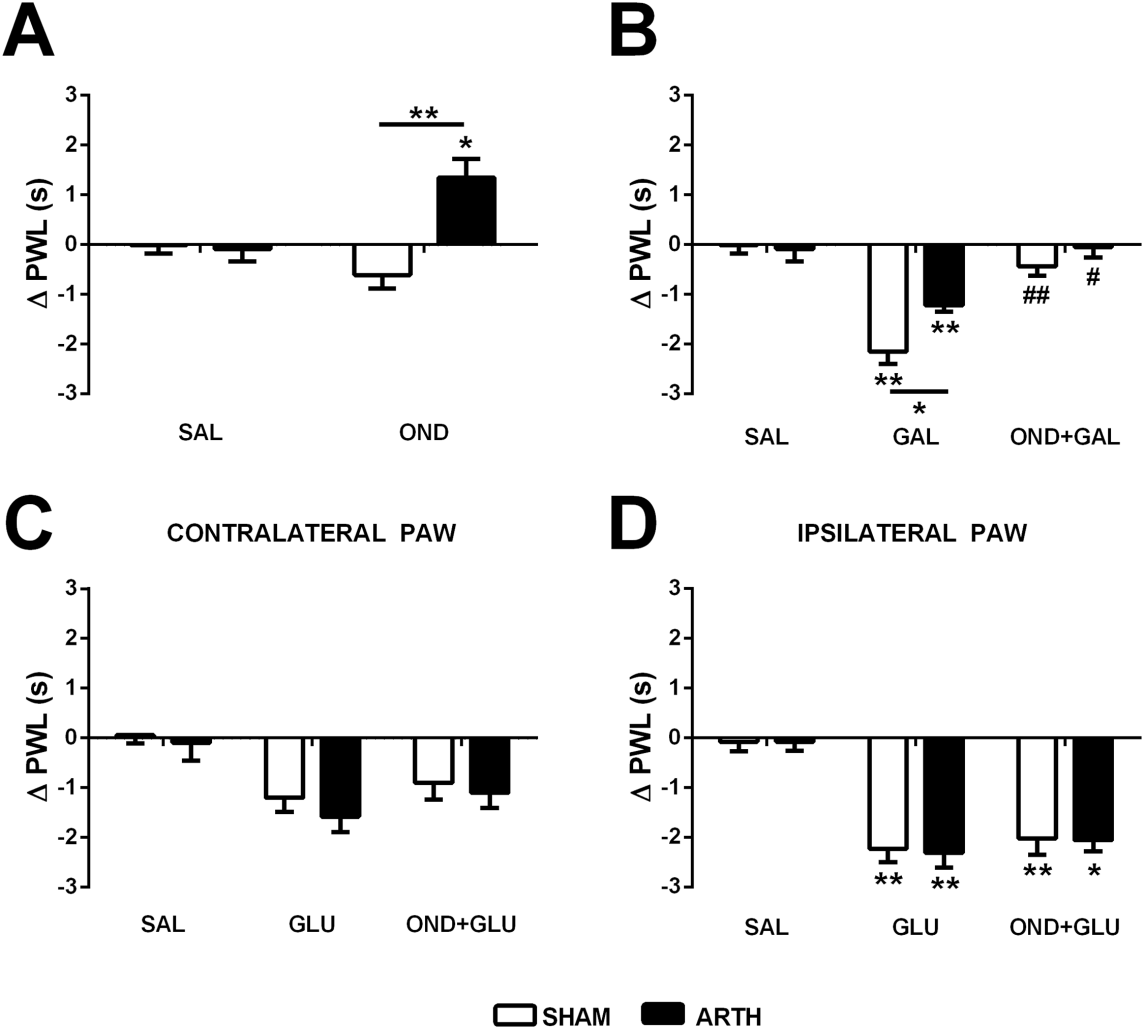
Behavioural evaluation of paw-withdrawal latencies (PWL) after the drug microinjection in the dorsomedial nucleus of the hypothalamus (DMH), medullary dorsal reticular nucleus (DRt) and spinal cord of control (SHAM) and arthritic (ARTH) animals. **(A)** PWL after spinal administration of ondansetron (OND). **(B)** PWL after administration of galanin (GAL) in the DMH or the combination of GAL in the DMH and OND in the spinal cord. **(C)** PWL in the contralateral **(D)** and ipsilateral hind paw after glutamate (GLU) administration in the DRt or after the combined administration of OND in the spinal cord and GLU in the DRt. Saline (SAL) injections were used as controls. Graphs show mean PWL + SEM (n_SHAM_ = 7; n_ARTH_ = 5). *P<0.05, **P<0.01: Comparison of the drug effect with the effect of SAL within the same experimental group; ^**#**^ *P*<0.05, ^**##**^ *P*<0.01: Comparison of the effect of drug combination with the corresponding drug alone (t-test with a Bonferroni correction for multiple comparisons).

To study the tonic action of the spinal 5HT_3_R on nociception in SHAM and ARTH animals, the PWL was evaluated after injecting OND, a 5HT_3_R antagonist, intrathecally at the lumbar spinal cord level. PWL changes after OND administration varied with the experimental group (interaction between drug treatment and experimental group *F*_2,10_ = 14.638, *P* = 0.003). *Post hoc* tests indicated that PWL was increased after spinal OND treatment in ARTH animals alone (Fig 3A).

To investigate a possible role of the spinal 5HT_3_R in the mediation of the pronociceptive effect of GAL in the DMH, PWL was assessed after GAL microinjection in the DMH following spinal administration of OND. PWL was significantly altered by drug administrations (main effect of drug: *F*_2,20_ = 35.977, *P*<0.001). *Post hoc* tests indicated that PWL was decreased after GAL microinjection in the DMH; this hyperalgesic effect was stronger in the SHAM than ARTH group and it was reversed by spinal administration of OND in both SHAM and ARTH animals (Fig 3B).

To study a possible role of the spinal 5HT_3_R in the mediation of the pronociceptive effect of the DRt, PWL was assessed after activation of the (right) DRt by GLU and inhibition of the spinal 5HT_3_R by OND. In the contralateral (left) paw, PWL was significantly altered after drug administrations (main effect of drug: *F*_2,20_ = 8.672, *P =* 0.002), although *post hoc* tests did not indicate any difference between treatment groups (Fig 3C). In the ipsilateral (right) paw, the PWL was significantly altered after drug administrations (main effect of drug: *F*_2,20_ = 35.277, *P*<0.001). *Post hoc* tests indicated that PWL was decreased after GLU administration in the DRt and that OND in the spinal cord failed to reverse this pronociceptive effect (Fig 3D).

### Electrophysiological analysis

#### Arthritis increases the activity of spinal dorsal horn neurones

**Fig 4:**
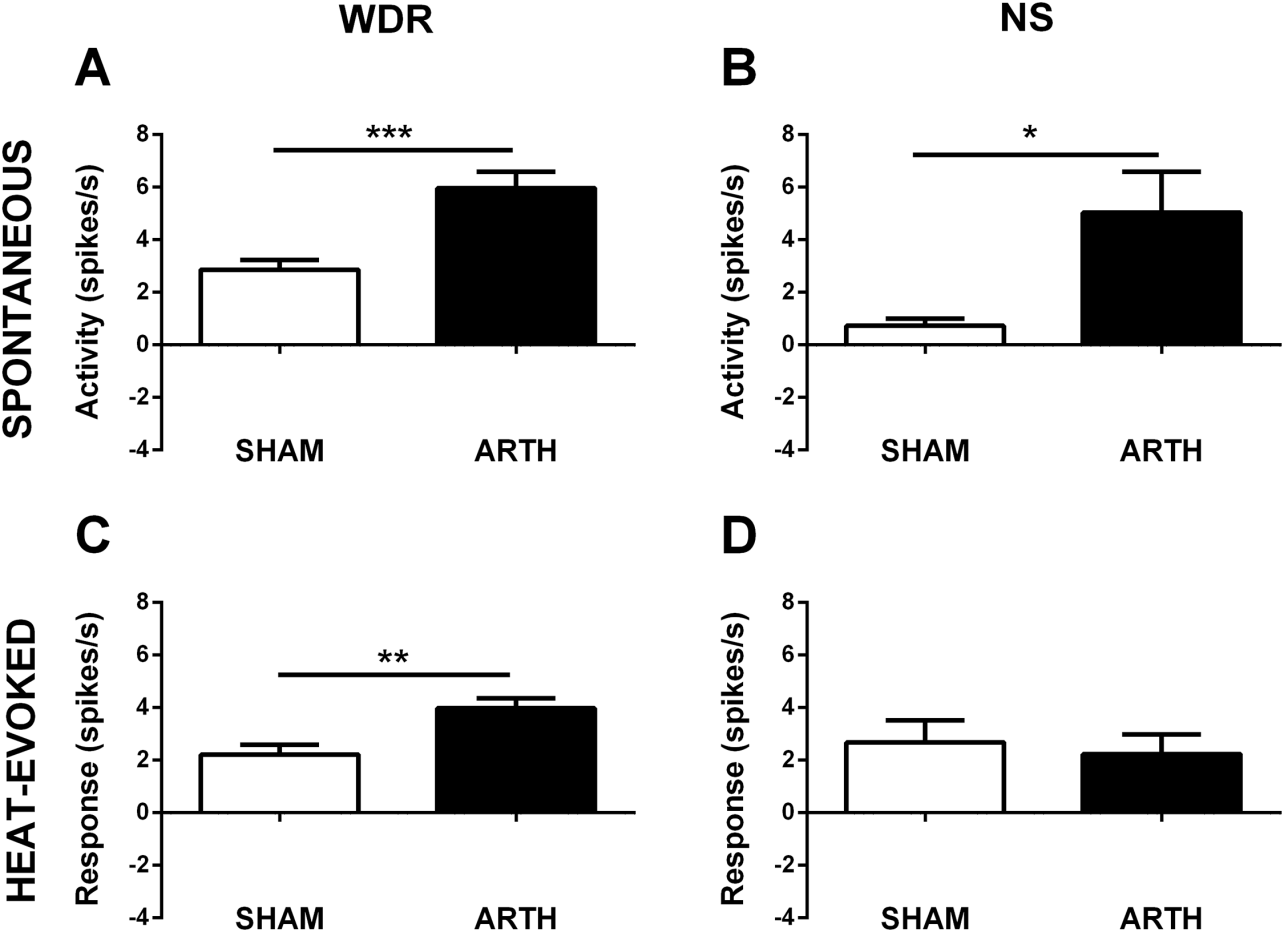
Baseline activity of spinal dorsal horn wide-dynamic-range (WDR) and nociceptive-specific (NS) neurons of control (SHAM) and arthritic (ARTH) animals. Spontaneous activity (WDR:**A**, NS:**B**) and heat-evoked responses (54°C; WDR:**C**, NS:**D**) in ARTH and SHAM groups. Graphs show mean neuronal activity + SEM (WDR: n_SHAM_ = 40; n_ARTH_ = 68; NS: n_SHAM_ = 10; n_ARTH_ = 12). *P<0.05, **P<0.01, ***P<0.001 (t-test for unpaired data).

To evaluate the effect of arthritis upon SDH neurones, discharge properties of WDR and NS neurones were assessed in SHAM and ARTH animals. ARTH induced an increase in the spontaneous activity of WDR (*t*_106_ = 3.570, *P*<0.001) (Fig 4A) and NS (*t*_20_ = 3.570, *P*<0.001) (Fig 4B) neurones. In parallel, ARTH increased heat-evoked activity of WDR (*t*_106_ = 3.024, *P =* 0.003) (Fig 4C) but not NS (*t*_20_ = 0.3904, *P =* 0.700) (Fig 4D) neurones.

### GAL in the DMH increases activity of spinal dorsal horn neurones

**Fig 5:**
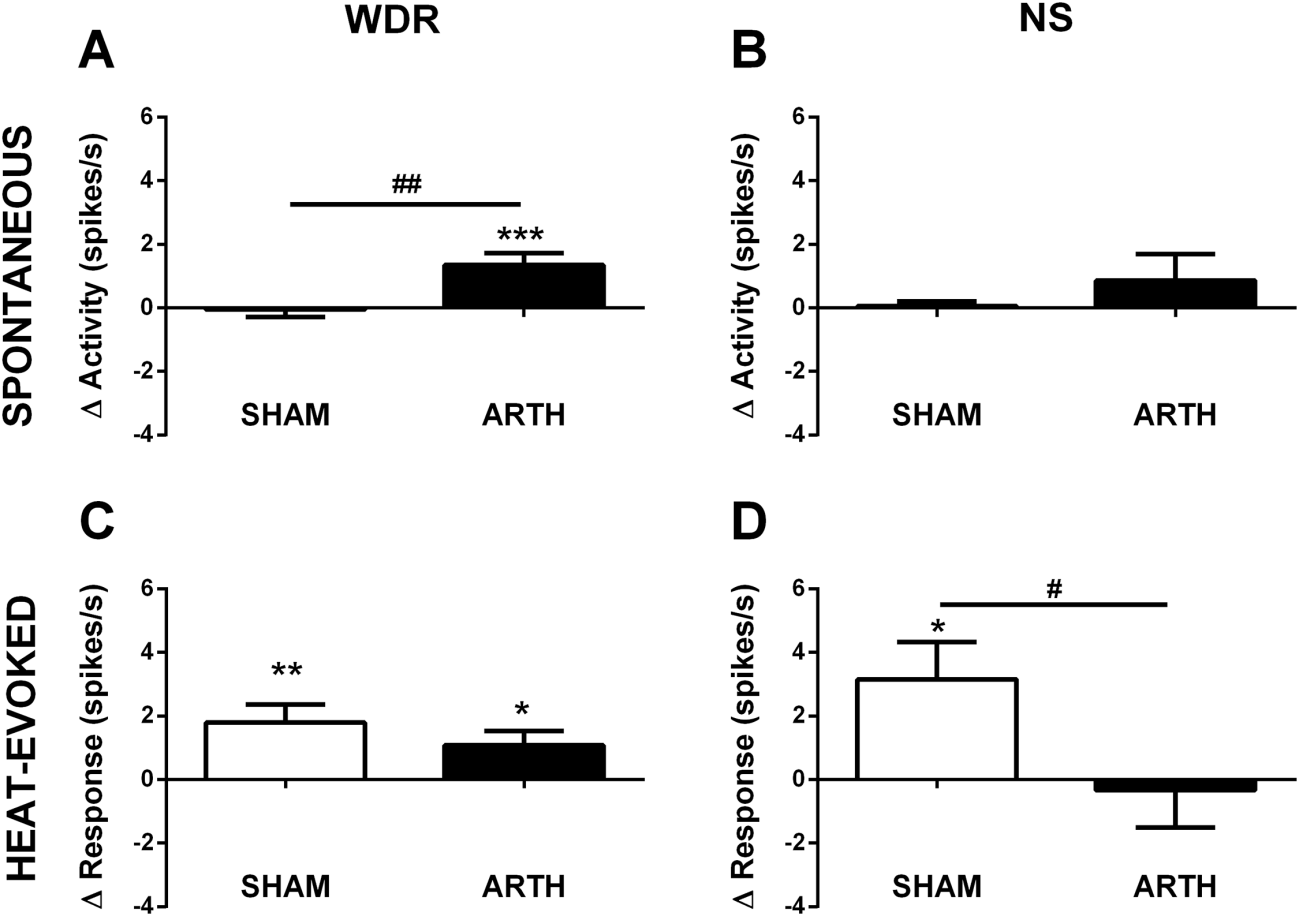
Effect of galanin (GAL) injection in dorsomedial nucleus of the hypothalamus (DMH) on spontaneous and stimulus-evoked activity in spinal dorsal horn wide-dynamic-range (WDR) and nociceptive specific (NS) neurones of control (SHAM) and arthritic (ARTH) animals. Mean changes in spontaneous (WDR:**A**, NS:**B**) and heat-evoked (54°C; WDR:**C**, NS:**D**) activities of WDR and NS neurones are shown 20 min after GAL administration in the DMH. Δ Activity—represents the difference between spontaneous activity 20 min after GAL injection in the DMH and the spontaneous activity before GAL administration (baseline). Δ Response—represents the difference between neuronal response 20 min after GAL injection in the DMH and the response before GAL administration (baseline). Δ Activity or Δ response values > 0 represent GAL-induced increase in the neuronal activity or response. Graphs show mean activity + SEM (WDR: n_SHAM_ = 40; n_ARTH_ = 68; NS: n_SHAM_ = 10; n_ARTH_ = 12). *P<0.05, **P<0.01, ***P<0.001: Comparison of the effect of GAL with the corresponding pre-injection value (one sample t-test; pre-injection value = 0). ^**#**^ *P*<0.05, ^**##**^ *P*<0.01: Comparison of the effect of GAL between SHAM and ARTH groups (t-test for unpaired data).

To study the effect of GAL administration in the DMH upon SDH neurones, spontaneous and heat-evoked activities of WDR and NS neurones were assessed in SHAM and ARTH animals. Comparisons with the corresponding pre-injection values indicated that GAL in the DMH induced an increase in spontaneous activity of WDR neurones in ARTH (*t*_67_ = 3.521, *P<*0.001; n_WDR_ = 68) but not SHAM (*t*_39_ = 0.238, *P =* 0.813; n_WDR_ = 40) animals (Fig 5A). Spontaneous activity of NS neurones was not altered by the administration of GAL in the DMH either in SHAM (*t*_9_ = 0.382, *P = 0*.711; n_NS_ = 10) or ARTH (*t*_11_ = 1.038, *P =* 0.322; n_NS_ = 12) animals (Fig 5B).

GAL microinjection in the DMH induced an increase in heat-evoked activity of WDR neurones in both SHAM (*t*_39_ = 3.137, *P =* 0.003) and ARTH (*t*_67_ = 2.393, *P =* 0.020) animals (Fig 5C), while it increased heat-evoked activity of NS neurones in SHAM (*t*_9_ = 2.699, *P* = 0.024) but not ARTH (*t*_11_ = 0.290, *P =* 0.777) animals (Fig 5D).

### GLU injection in the DRt increases activity of spinal dorsal horn neurones

**Fig 6:**
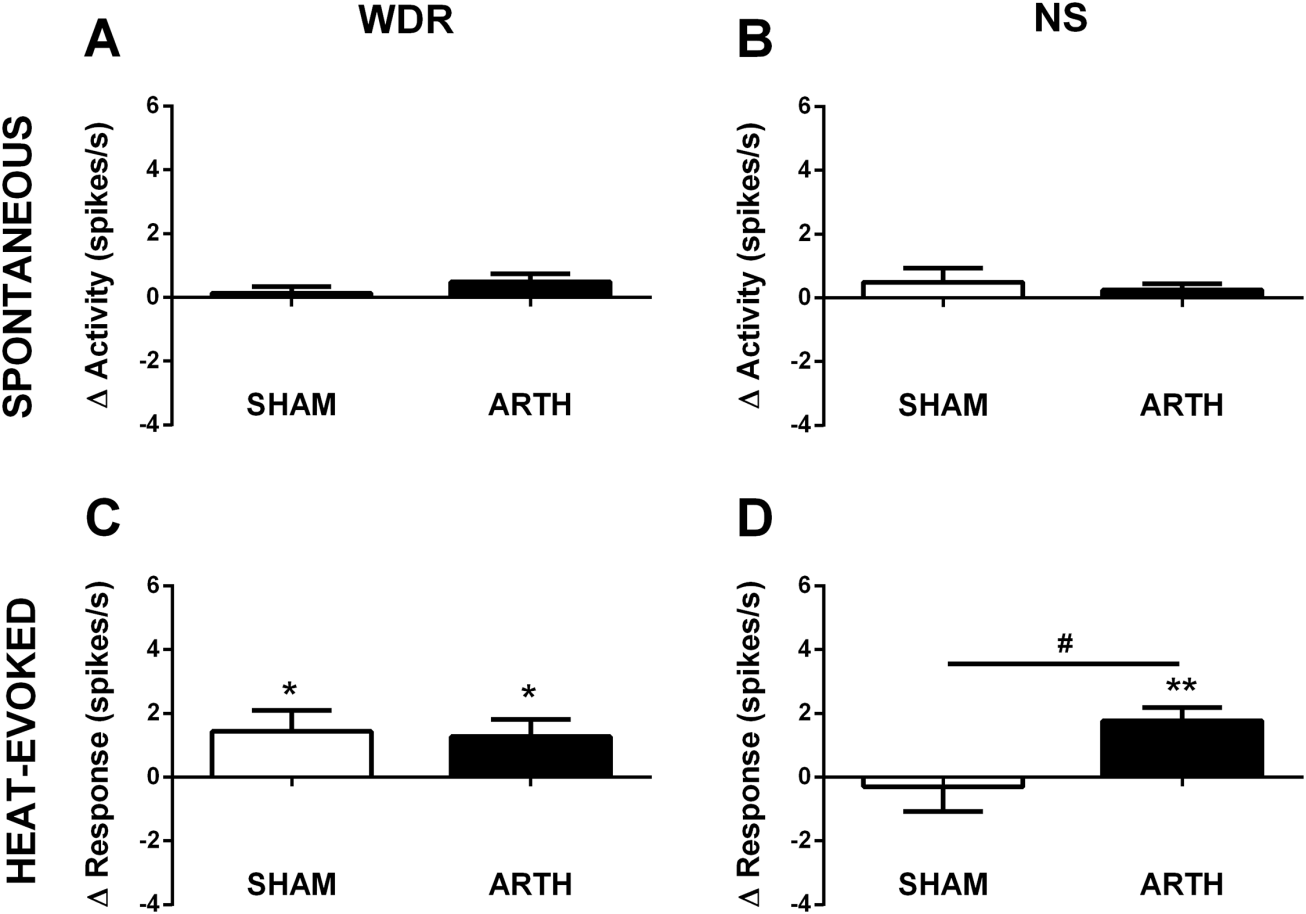
Effect of glutamate (GLU) injection in the medullary dorsal reticular nucleus (DRt) on activity of spinal dorsal horn wide-dynamic-range (WDR) and nociceptive specific (NS) neurons in control (SHAM) and arthritic (ARTH) animals. Mean changes in spontaneous (WDR:**A**, NS:**B**) and heat-evoked (54°C. WDR:**C**, NS:**D**) activities of WDR and NS neurones are shown 1 min after GLU administration in the DRt. Δ Activity—represents the difference between spontaneous activity 1 min after GLU injection in the DRt and the spontaneous activity before GLU administration (baseline). Δ Response—represents the difference between neuronal response 1 min after GLU injection in the DRt and the response before GLU administration (baseline). Δ Activity or Δ response values > 0 represent GAL-induced increase in the neuronal activity or response. Graphs show mean changes in neuronal activity + SEM (WDR: n_SHAM_ = 31; n_ARTH_ = 53; NS: n_SHAM_ = 7; n_ARTH_ = 9). *P<0.05, **P<0.01: Comparison of the effect of GLU with the corresponding pre-injection value (one sample t-test; pre-injection value = 0). ^**#**^ *P*<0.05: Comparison of the effect of GLU between SHAM and ARTH groups (t-test for unpaired data).

To study the effect of GLU administration in the DRt upon activity of SDH neurones, spontaneous and heat-evoked activities of WDR (n_SHAM_ = 31; n_ARTH_ = 53) and NS (n_SHAM_ = 7; n_ARTH_ = 9) neurones were assessed in SHAM and ARTH animals. GLU administration in the DRt did not alter spontaneous activity of WDR neurones in SHAM (*t*_30_ = 0.727, *P =* 0.473) or ARTH (*t*_52_ = 1.942, *P =* 0.058) animals (Fig 6A). Similarly, GLU in the DRt did not alter spontaneous activity of NS neurones in SHAM (*t*_6_ = 1.146, *P =* 0.295) or ARTH (*t*_8_ = 1.346, *P =* 0.215) animals (Fig 6B). In contrast, GLU in the DRt increased heat stimulation-evoked response of WDR neurones in both SHAM (*t*_30_ = 2.184, *P =* 0.037) and ARTH (*t*_52_ = 2.306, *P =* 0.025) animals (Fig 6C) while it induced an increase in heat-evoked response of NS neurones only in ARTH animals (*t*_8_ = 4.330, *P =* 0.003) (Fig 6D).

## Discussion

In line with our recent results [6], GAL in the DMH induced behavioural hyperalgesia in monoarthritic (ARTH) as well as control (SHAM) animals. Importantly, here we show that two independent descending pathways, one relaying in the DRt and the other acting on the spinal 5HT_3_Rs, are among relays contributing to the GAL-induced facilitation of pain behaviour. Both of these descending pronociceptive pathways were tonically active in the ARTH but not SHAM animals.

The utility of in vivo preclinical models is unquestionable but its applicability to the study of human diseases remains debatable. In what concerns arthritis most animal models reflect only a small part of the human disorder such as the inflammatory, mechanical or degenerative components [38]. In addition and according to Vincent et al. (2012) [39] animal models either model painful behaviour (chemical and surgical models) or cartilage degeneration (spontaneous and surgical models) with none mirroring accurately its pathogenic mechanisms or response to treatment.

Recently many groups chose to use chemical models such as the intra-articular injection of monoiodoacetate that induces acute cartilage degradation and joint pain although its effects are too extensive when compared to early stage human OA [40,41]. Also considerably studied are surgical/traumatic models, such as the medial meniscus transection, considered relevant in the context of impact upon articular structures and pain but limited in terms of the inflammatory component of OA [40,41].

In this work we opted to inject a mixture of kaolin and carrageenan in the right knee. As shown in a previous work by Amorim et al. (2014) [42], kaolin/carrageenan induce a slowly developing use-dependent degeneration of the knee joint translated as oedema, inflammatory reaction of articular structures, cartilage thinning, focal disorganization of chondrocytes, sclerotic bone and development of cysts, changes concomitant with the development of human OA grade 4. In addition, animals also develop mechanical hyperalgesia and emotional impairments, both important features of the human condition. The advantages of this model over others also include a slower onset and progression and its restriction to a single joint.

### The GAL/DMH-DRt facilitatory pathway

Recently, we showed that activation of the DMH by GLU [8] or GAL [6] induced behavioural hyperalgesia. Interestingly, while the pronociceptive effect of GLU in the DMH of healthy controls was mediated by the RVM [8], the circuits mediating GAL/DMH-induced descending facilitation remained unclear [6]. Moreover, the facilitatory pathway activated by GLU in the DMH was impaired in experimental monoarthritis [8], while behavioural hyperalgesia could still be evoked by GAL in the DMH of monoarthritic animals [6]. These results suggest that at least partly different circuits were mediating the descending facilitatory effects induced by GLU and GAL in the DMH.

By activating the DMH with GAL and inhibiting the DRt with LIDO, we demonstrated that the GAL/DMH-induced descending facilitation was, at least in part, mediated by the DRt, since local blocking of the DRt prevented the GAL/DMH-induced behavioural hyperalgesia. These results are in accordance with several earlier studies showing a facilitatory role of the DRt in nociceptive modulation [11,14–19]. Indeed, Almeida and colleagues [15] demonstrated that activation of the DRt by GLU decreased tail-flick latency and, conversely, lesion of the DRt increased response latencies in the tail-flick test, hot-plate test and the acute and inflammatory phases of the formalin test [14,15]. In addition, the DMH does not project directly to the SDH [9], but projects to the DRt [9,10], which indicates that DRt neurones can mediate the descending [43,44] pain modulatory action of the DMH.

In the present study, inhibition of the DRt induced an increase in PWL in ARTH but not SHAM animals indicating that the DRt contributes to tonic facilitation of nociception in monoarthritis. Enhanced tonic descending facilitation from the DRt is in accordance with earlier reports showing an increase in c-Fos expression and metabolic activity of DRt in experimental monoarthritis [45,46]. Moreover, since the DRt projects directly to the superficial dorsal horn [43,44], the increase in spontaneous activity of spinal WDR and NS neurones in ARTH animals, a finding commonly reported in several models of neuropathic and inflammatory pain [17,47,48], might partly be a consequence of enhanced DRt-mediated tonic facilitation.

Interestingly, our data showed that in ARTH animals the facilitatory effect relayed by the DRt descends predominantly ipsilaterally as evidenced e.g. by the predominantly ipsilateral effects of the LIDO-induced inhibition of the DRt on PWL. Although DRt laterality has not often been studied, it was previously demonstrated that in the formalin test, the lesion of the ipsilateral DRt decreased nociceptive phase 1 and 2 behaviours. In contrast, contralateral lesion of the DRt only affected phase 2 behaviour in the formalin test and was accompanied by a decrease of c-Fos positive cells in the ipsilateral SDH laminae I-II and IV-V [14].

### The GAL/DMH-5HT_3_R facilitatory pathway

The existence of descending serotonergic circuits that modulate nociceptive transmission is widely known [49,50]. Serotonergic pathways can either inhibit or enhance nociception depending of the receptor subtypes that are being expressed and activated in the SDH [49]. Here, we focused on 5HT_3_Rs, as they are mainly expressed on small diameter afferent terminals of the superficial dorsal [51,52]. Spinal 5HT_3_Rs are known to participate in modulation of nociception, although their role in nociceptive processing has been not completely clear since both antinociceptive and pronociceptive effects have been reported [25,53–57].

We found that OND, a 5HT_3_R antagonist injected in the lumbar spinal cord, increased heat-evoked PWLs in ARTH but not SHAM control animals. This finding indicates that the spinal 5-HT_3_R tonically facilitates nociception in experimental monoarthritis. This result is in accordance with earlier results [58] showing that blocking the spinal 5HT_3_R had hardly any effect on nociception in healthy animals while it decreased nociception in inflamed [26] or nerve-injured animals [35], and reduced nociceptive (second phase) behaviour in the formalin test [26,59]. Additionally, neuropathic hypersensitivity was profoundly reduced in 5HT_3_R-depleted rats [27]. Conversely, the intrathecal administration of a 5HT_3_R agonist facilitated nociceptive behaviours in the late phase of the formalin test [26]. In humans, intravenous administration of OND significantly decreased pain scores in patients with chronic neuropathic pain, again suggesting that serotonergic circuits acting on the 5HT_3_R promote tonic pain facilitation in neuropathy [60]. Together these data suggest 5HT_3_Rs facilitate nociception in chronic neuropathic and inflammatory disorders.

Among several supraspinal centres that project to the spinal dorsal horn, the RVM, in particular the nucleus raphe magnus, has been proposed to play a key role in descending bidirectional modulation of nociception both in physiological conditions [61,62] and chronic pain states [50,63,64]. In neuropathic conditions, descending facilitatory action of RVM appears to be essential for the maintenance, but not the initiation, of chronic pain [65].

Our recent electrophysiological results suggest that pronociceptive On-like or antinociceptive Off-like cells in the RVM do not mediate the descending pronociceptive action induced by GAL in the DMH [6]. It should be noted that RVM On- and Off-cells are non-serotonergic cells while a subpopulation of RVM Neutral-cells that do not respond to peripheral noxious stimulation have been shown to be 5-HT immunoreactive [24]. Taking into account that the RVM is a major source of serotoninergic innervation of the SDH [49] and that blocking the spinal 5HT_3_Rs reversed the pronociceptive action induced by GAL in the DMH, it may be proposed that serotonergic RVM cells (a subpopulation of RVM Neutral cells) provide a relay to the GAL/DMH-induced descending facilitation. In our earlier study, GAL in the DMH increased c-fos expression in the RVM [6]. This finding together with an accompanying electrophysiological result showing no corresponding change in the discharge of non-serotonergic RVM On- or Off-like cells supports the proposal that serotonergic RVM cells may provide a relay for descending action of GAL in the DMH, although verification of this hypothesis by electrophysiological recordings of the subpopulation of serotonergic RVM Neutral-cells is still missing [6].

### Spinal neuronal correlates of behavioural findings

In the spinal dorsal horn, increased spontaneous activity of both WDR and NS neurones was the most prominent neuronal correlate of experimental arthritis, an effect possibly associated with sustained arthritic pain. GAL in the DMH increased stimulus-evoked responses of spinal WDR neurones both in SHAM and ARTH animals, while spontaneous activity of WDR neurones was increased only in the ARTH group. In contrast, GAL in the DMH failed to have a significant influence on the activity of spinal NS neurones, except for an increase of the heat-evoked response in the SHAM group. Glutamatergic DRt activation indicated that this nucleus facilitates heat-evoked but not spontaneous activity of nociceptive spinal dorsal horn neurones. Moreover, while the heat-evoked response of WDR neurons was facilitated by the DRt both in SHAM and ARTH animals, the heat-evoked response of NS neurones was increased by glutamatergic DRt activation only in the ARTH group.

The facilitation of heat-induced pain behaviour following GAL treatment of the DMH or glutamate treatment of the DRt correlated most consistently with an increase of heat-evoked responses of spinal WDR rather than NS neurones both in SHAM and ARTH groups. This finding is in line with earlier reports indicating that spinal WDR neurones are involved in encoding of nociceptive signals [66], whereas NS neurones may have a predominant role in other functions, such as feedback-inhibition of pain or control of autonomic responses [37]. It should be noted that in the present study spinal dorsal horn WDR and NS neurones were not divided into further subclasses based e.g. on their projections or neurochemical properties. It remains to be studied whether the correlations of behavioural findings with the spinal neuronal response varies with the projection or neurochemistry of the subpopulation of WDR or NS neurones.

## Conclusions

GAL in the DMH activates in parallel two descending pronociceptive pathways one of which relays in the DRt and the other one in serotoninergic brainstem neurons that have descending projections promoting nociception through action on the spinal 5HT_3_R. Both of these descending pronociceptive pathways are tonically active in monoarthritic but not healthy control animals.

## Abbreviations

5HT_3_R: serotonin type 3 receptor
AP: anteroposterior
ARTH: arthritic animals
DMH: dorsomedial nucleus of the hypothalamus
DRt: dorsal reticular nucleus
DV: dorsoventral
GAL: galanin
GLU: glutamate
i.t.: intrathecal
LIDO: lidocaine
LM: lateromedial
LWT: limb withdrawal threshold
NS: nociceptive specific neurones
OND: ondansetron
PWL: paw withdrawal latency
RVM: rostral ventromedial medulla
SAL: saline solution
SDH: spinal dorsal horn
SHAM: control animals
WDR: wide dynamic range neurones
